# Temporal contact patterns and the implications for predicting superspreaders and planning of targeted outbreak control

**DOI:** 10.1098/rsif.2024.0358

**Published:** 2024-12-18

**Authors:** Rachael Pung, Josh A. Firth, Timothy W. Russell, Tim Rogers, Vernon J. Lee, Adam J. Kucharski

**Affiliations:** ^1^ Centre for the Mathematical Modelling of Infectious Diseases, London School of Hygiene and Tropical Medicine, London, UK; ^2^ Department of Infectious Disease Epidemiology, London School of Hygiene and Tropical Medicine, London, UK; ^3^ Ministry of Health, Singapore; ^4^ Department of Biology, University of Oxford, Oxford, UK; ^5^ Merton College, University of Oxford, Oxford, UK; ^6^ Faculty of Biological Sciences, University of Leeds, Leeds, UK; ^7^ Department of Mathematical Sciences, University of Bath, Bath, UK; ^8^ National Centre for Infectious Diseases, Singapore; ^9^ Saw Swee Hock School of Public Health, National University of Singapore, Singapore

**Keywords:** temporal network, outbreak control, superspreader, superspreading

## Abstract

Directly transmitted infectious diseases spread through social contacts that change over time, but outbreak models typically make simplifying assumptions about network structure and dynamics. To assess how common assumptions relate to real-world interactions, we analysed 11 networks from five settings and developed metrics, capturing crucial epidemiological features of these networks. We developed a novel metric, the ‘retention index’, to characterize the distribution of retained contacts over consecutive time steps relative to fully static and dynamic networks. In workplaces and schools, contacts in the same department formed most of the retained contacts. In contrast, no clear contact type dominated the retained contacts in hospitals, thus reducing overall risk of disease introduction would be more effective than control targeted at departments. We estimated the contacts repetition over multiple days and showed that simple resource planning models overestimate the number of unique contacts by 20%–70%. We distinguished the difference between ‘superspreader’ and infectious individuals driving ‘superspreading events’ by measuring how often the individual represents the top 80% of contacts in the time steps over the study duration. We showed an inherent difficulty in identifying ‘superspreaders’ reliably: less than 20% of the individuals in most settings were highly connected for multiple time steps.

## 1. Background

**Figure 1.**
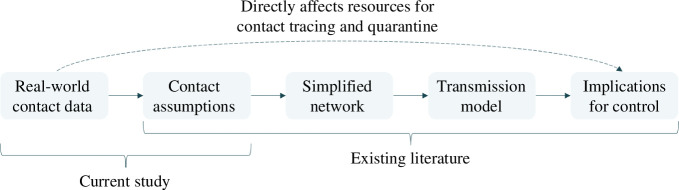
Different components of contact network studies and how they influence outbreak control measures.

Directly transmitted infections spread through human social contacts, but the dynamic and often high-dimensional nature of these networks has historically made them difficult to measure and interpret. As a result, epidemic models often implicitly approximate complex dynamic networks with simpler contact processes, including static networks [1,2], branching processes [3] and compartmental models [4]. These relatively simpler models of disease transmission have been well studied (figure 1), but it remains unclear how they compare with real-life temporal social networks, which exhibit a mix of repeated and occasional contacts [5,6]. As such, the assumptions in these simpler models could bias model outputs that are crucial for epidemic planning and response, from estimating the required resources for contact tracing and testing programmes to assessing the impact of social distancing measures and vaccine coverage [7–9].

There has been recent progress in the collection of dynamic contact network data via proximity sensors [10,11] or mobile devices [12]. The automated nature of such data collection enabled large-scale deployment for contact tracing during the COVID-19 pandemic [11,13]. These devices work by exchanging radio frequency identification (RFID) signals within a calibrated distance, enabling us to monitor contacts and map the emerging network structure. This can—in theory—enable us to interpret the transmission process on temporal networks. However, in practice, most studies still tend to simplify the temporal network structure by extending static network properties, which depend on characteristics such as population sizes [5], making it hard to compare findings across studies. Furthermore, it can be challenging to tease out the effects of different network features on the transmission dynamics in models [5,14,15]. Finally, temporal contact data in some studies were collected through self-reported contact diaries, which may be prone to recall bias [6,14,16]. With the extensive data collected from automated devices, this is increasingly an opportunity to better compare contact structures and, hence, the implications for key transmission processes.

Using real-world temporal social data from over 4 million contact events collected across five settings (cruises, community, schools, hospital and workplaces), we quantified the impact of dynamic contacts on key epidemiological metrics driving person-to-person transmission across these varied social settings. As well as examining the range of bias in the number of unique contacts introduced by common simplifying assumptions, we identify the extent to which it is possible to identify individuals linked to superspreading events reliably. To characterize time-varying properties of the real-life networks, we developed a new metric—the retention index—that allows complex dynamic networks to be summarized and compared in an epidemiologically meaningful manner.

## 2. Methods

### 2.1. Temporal contact network data

We collated temporal contact network data from previously published studies across different settings, with contacts recorded using proximity sensors or mobile devices (table 1 and electronic supplementary material, table S1). These devices were calibrated to record contacts between pairs of individuals within a specified radius on cruises and in a community or, alternatively face-to-face interactions in high schools, hospitals and workplaces. The radius approach is omnidirectional, while the face-to-face methods record a contact when the sensors face each other. For each network, we performed preliminary analysis to identify common types of contact, contact durations and delays before the next contact occurs between the same pair of individuals (table 1). Contact data from the cruises were recorded in 15 s intervals, while in all other networks, contacts were recorded in 5 min or 20 s intervals.

**Table 1.** Characteristics of real-world contact networks.

network setting	study date, observed days	types of contact	median contact duration (s)	median delay in contact (s)	remarks (references)
cruises, Singapore	Nov 2020, 3 d	P: passenger	900 for all four sailings	900 for all four sailings	COVID-19 restrictions on-board. Undirected network; refer to referenced study for detailed network plots [11]
	Nov 2020, 3 d	C: crew			
	Jan 2021, 3 d	P–P (same cabin)			
	Feb 2021, 3 d	P–P (different cabin)			
	(i.e. four sailings with two in Nov 2020)	C–C (same department)			
		C–C (different department)			
		P–C			
community, Haslemere, UK	Oct 2017, 3 d	household	300	600	No data before 07.00 and after 23.00. Directed network; refer to referenced study for detailed network plots [12]
		non-household			
high schools, Marseilles, France	Dec 2011, 4 d	classmates	20 for all three high school	140	No data over weekends. Directed network [16,17]
	Nov 2012, 7 d	non-classmates		120	
	Dec 2013, 5 d			100	
hospital, Lyon, France	Dec 2010, 5 d	same department	20	140	Directed network [18]
		different department			
workplaces, France	Jun 2013, 10d	same department	20 for both workplaces	220	No data over weekends. Directed network [19,20]
	2015, 10d	different department		120	

To analyse the network properties, we first needed to choose a timescale for defining a ‘contact’ within each dataset. In our main analysis, a contact is defined to occur within a time step if it lasts for at least the median contact duration for respective networks (table 1). For the cruise networks involving 1500−2000 individuals per sailing, the median contact duration was 15 min. With devices capable of omnidirectional signal detection, 20 000−50 000 contacts lasting at least 15 min per sailing were recorded (electronic supplementary material, table S1). For the school, hospital and workplace networks, the population size in respective networks was about 100–300 individuals. Furthermore, with devices capturing only face-to-face interactions, less than 100 contacts lasted for at least 15 min in each network (electronic supplementary material, table S1). Instead, the median contact duration was 20 s and contacts lasting for at least two 20 s intervals (i.e. 40 s) served as a proxy for continuous interaction. As such, we designed the main analysis based on the characteristics of each network and defined a valid contact to last for at least the median contact duration of the respective network.

In our main analysis, we set the length of the time step for each network based on the median delay in contact (table 1). For the high school, hospital and workplace networks, a small time step (e.g. 20 s) would result in few repeated contacts over consecutive time steps because the median delay between contact events was higher than the contact duration (table 1). As such, the main analysis considered the contact patterns based on time steps defined for each network. For sensitivity analysis, the time step was set at 15 min or 1 h and standardized across all networks. We also performed additional sensitivity analysis, assuming the directed contact networks in the non-cruise settings were undirected.

**Figure 2.**
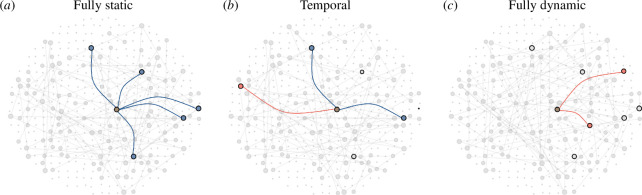
Contacts made by an individual of interest (brown, centre) in a single time step with contacts retained from the previous time step (blue), contacts that were not retained from the previous time step (grey with black outline) and new contacts in current time step (red) for (*a*) fully static, (*b*) temporal and (*c*) fully dynamic network.

At one theoretical extreme, networks may exhibit no variation over time, resulting in a static network, where the contacts remain the same over consecutive time steps; at the other extreme, we have fully dynamic networks, where every individual’s contacts are drawn randomly at each time step (figure 2). When simulating the fully dynamic network across consecutive time steps, we retained the degree distribution of each individual observed in a time step but randomly rewired their contacts. This ensures that the fully dynamic network has the same degree distribution as the static network of the same time step.

### 2.2. Contact retention

We defined a retained contact to occur when the same pair of individuals have recorded contact episodes over two consecutive time steps. To explore how contacts were retained and changed over time, we defined the distribution of the number of retained contacts, r, over consecutive time steps, t and t+1, in the network as follows:


(2.1) $$P\left(r_{t+1}\right)=\sum_{k_{t+1}=0}^{N-1}\sum_{k_{t}=0}^{N-1}P\left(r_{t+1}\;|\;k_{t},\;k_{t+1}\right)\;P\left(k_{t+1}\;|\;k_{t}\right)\;P\left(k_{t}\right),\;\;\;\;\;\;\;\;r_{t+1}\leq \;k_{t},\;k_{t+1},$$

where $r_{t+1}$ is the number of retained contacts over consecutive time steps t and t+1, $k_{t}$ is the number of contacts (i.e. degree) in time step t, and N is the number of individuals in a network. The maximum possible number of contacts an individual could make is N−1. For static or fully dynamic networks, where contacts are either fixed or made at random, $P\left(r_{t+1}\;|\;k_{t\;},\;k_{t+1}\right)$ of equation (2.1) is replaced with the binomial distribution as follows:


(2.2) P(rt+1)=∑kt+1=0N−1∑kt=0N−1(krt+1)prt+1(1−p)kt−rt+1 P(kt+1 | kt) P(kt),

where k is the minimum of $k_{t}$ and $k_{t+1}$ and p is the binomial probability of preserving a contact between a pair of individuals. For static networks, p=1 and the binomial term in equation (2.2) equals 1 when $k=r_{t+1}$ and 0 otherwise. Furthermore, individuals with k contacts in time step t will have the same number of contacts at time step t+1 as shown in equation (2.3). Overall, equation (2.2) simplifies into equation (2.4) as follows; illustrating that the degree distribution in time step t reflects the distribution of contacts retained over the next time step t+1:


(2.3) $$\begin{array}{l} P\left(k_{t+1}|k_{t}\right)=1,\;\;\;\;\;\;\;\;k_{t+1}=k_{t}\;\\ \;\;\;\;\;\;\;\;\;\;\;\;\;\;\;\;=\;0,\;\;\;\;\;\;\;\;k_{t+1}\neq k_{t} \end{array},$$

(2.4) $$P\left(r_{t+1}\right)=P\left(k_{t}\right).$$

For fully dynamic networks with randomly made links, $p=\frac{k_{t+1}}{N-1}$ and equation (2.2) is expressed as follows:


(2.5) $$P\left(k_{t+1}\;|\;k_{t}\right)\;=P\left(k_{t+1}\right),$$

(2.6) P(rt+1)=∑kt+1=0N−1∑kt=0N−1(krt+1)prt+1(1−p)kt−rt+1 P(kt+1) P(kt).

By definition, we expect the highest mean number of retained contacts to be observed in static networks, ${\overset{-}{r}}_{stat},$ and the lowest in fully dynamic networks, ${\overset{-}{r}}_{dyna}$ (figure 2). To quantify the mean number of retained contacts in our collated temporal networks, ${\overset{-}{r}}_{temp}$, we computed a scaled metric, defined as the ‘retention index’, as follows


(2.7) $$\overset{-}{r}=\frac{{\overset{-}{r}}_{temp}-{\overset{-}{r}}_{dyna}}{{\overset{-}{r}}_{stat}-{\overset{-}{r}}_{dyna}}.$$

This metric (retention index) provides a standardized measure of where a network lies between the two theoretical extremes. If $\overset{-}{r}\to 1$, the temporal network reflects a fully static (and hence fully predictable) structure; when $\overset{-}{r}\to 0$, the temporal network reflects a fully dynamic (and hence non-predictable) structure.

### 2.3. Epidemiological metrics

If contacts are retained over consecutive time steps, it will result in a longer duration of continuous contact and, hence, a higher risk of transmission. Under the assumption that infection does not change the individual’s contact patterns (e.g. for an infection that exhibits substantial asymptomatic or pre-symptomatic transmission), clustering of retained contacts would also limit further disease transmission by an infector if the contact is already infected. To identify predictors of contact retention over consecutive time steps, we estimated the proportion of repeated contacts occurring for each type of contact (table 1). Besides evaluating the retention of contacts over consecutive time steps, we can also evaluate the repetition of contacts over different days by estimating the frequency distribution of contact encounters in days among all the contact pairs.

We also assessed the bias introduced when assuming independence of contacts over the days. To do this, we estimated the difference between the cumulative unique contacts from the start of the study to the day of interest, and the sum of unique contacts each day from the start to the day of interest. The latter could be equal to or greater than the former as there could be repeated counting of unique contacts over the days of observation. For example, we have three unique contact pairs in day 1 (A–B, C–D, E–F) and three unique contact pairs in day 2 (A–B, G–H, I–J). The cumulative number of unique contact pairs from day 1 to day 2 is five, while the sum of unique contacts each day from day 1 to day 2 is six. In most contact studies, the number of contacts made per day was typically reported but not the number of repeated contacts made over the days. Including information on repeated contacts can enhance the estimation of resources required for contact tracing and quarantine during an outbreak. In order to generalize the findings across different studies with different population sizes, we estimated this difference as a proportion of the population size (i.e. relative difference).

### 2.4. Extent of superspreaders and superspreading events

We defined potential ‘superspreaders’ as individuals frequently identified to account for the top 80% of the contacts made or contact duration over the observed period (see example in the next paragraph). We also define potential ‘superspreading events’ to be transmission driven by individuals less frequently identified to account for the top 80% of the contacts or contact duration over the observed period. The latter group of individuals typically forms few contacts. However, for a small proportion of the time, they have many or prolonged contacts and could disproportionately account for many transmission events in that time if they were infectious [21,22]. The objective of this section is to determine our ability to predict the classification of an individual (superspreader versus superspreading events) at any point in time given the observations from a fixed time period in respective real-world networks.

In each time step, we identified the individuals accounting for the top 80% of contacts or contact duration (i.e. highly connected individuals). The minimum and maximum proportion of time steps that an individual was identified in this top group could range between 0 and 1. For each incremental proportion of time steps, we estimated the proportion of the population identified for the corresponding time steps, $\rho_{s},$ as follows:


(2.8) $$\rho_{s}=\;\frac{\sum_{0}^{s}n}{N},$$

where $\sum_{0}^{s}n$ is the cumulative number of individuals who accounted for the top 80% of contacts or contact duration for at most s out of S time steps over the study period and N is the number of individuals in a network. When s=S, $\rho_{S}$ thus evaluates to 1.

To illustrate the extent of transmission events driven by superspreader or superspreading dynamics, we plot the cumulative proportion of the population identified for at least a given proportion of time. For example, we might identify a certain proportion of the population to be highly connected in at least half of the number of observed time steps (i.e. $\rho_{S}-\rho_{S/2}$). In this example, we could label this group as ‘superspreaders’. On the other hand, we might identify a certain proportion of the population to be highly connected but only in less than a quarter of the number of observed time steps (i.e. $\rho_{S/4}$). We could label this group as individuals who drive ‘superspreading events’. Individuals who are highly connected for a quarter to half the number of observed time steps lie between the regions of ‘superspreader’ and ‘superspreading event’. These cut-offs for ‘superspreaders’ and ‘superspreading events’, while arbitrary, serve as a starting point to categorize the level of connectivity of individuals and their risk of spreading infectious diseases.

To provide context of how the real-world networks in our main results compare with static and fully dynamic networks, we simulated a homogeneous and an overdispersed network over different time steps to estimate the above metrics. In a homogeneous network, expected 80% of the population accounts for 80% of the contacts (i.e. $p_{80}=$ 0.8), while in an overdispersed network, this is less than 80% of the population (in this study, we used 50%, i.e. $p_{80}=$ 0.5). For a static network, regardless of a homogeneous or an overdispersed network, the same proportion of the population was identified across all time steps by definition. For a fully dynamic network of varying time steps, the proportion of the population identified for each incremental proportion of time is approximately $p_{80}$ raised to the power of s, where s is the number of time steps corresponding to the proportion of time.

## 3. Results

### 3.1. Contact retention

**Figure 3.**
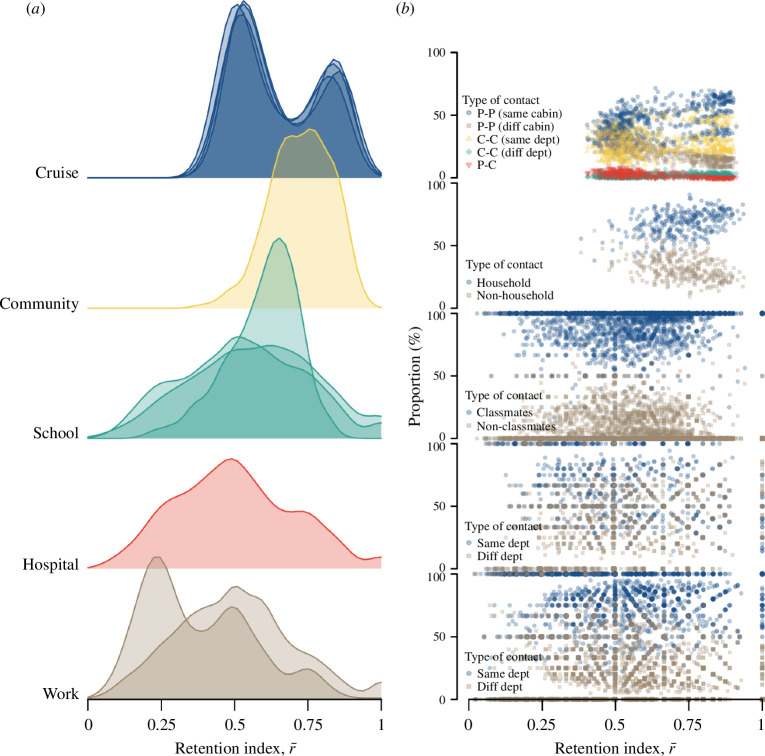
Contacts patterns in different network settings, (*a*) ridgeline plot showing distribution of contact retention index, $\overset{-}{r}$, over consecutive time steps and (*b*) proportion of the type of retained contacts for respective $\overset{-}{r}$.

We found considerable variation in the retention index $\overset{-}{r}$ across different networks and over time. For example, cruise networks exhibited an $\overset{-}{r}$ of 0.59 (interquartile range (IQR) 0.52−0.81). This study was conducted under strict COVID-19 physical distancing and social gathering restrictions on-board the cruises (figure 3*a*). As a result, most of the repeated contacts occurred among passengers who shared the same cabin and, hence, were in the same travelling group and crew members of the same department (figure 3*b*). We estimated an $\overset{-}{r}$ of less than 0.5 in only 12%−24% of the observed time steps for the four cruise sailings, indicating that in a given time period, contacts are much more likely to be retained rather than new contacts being made. Between 30% and 60% of these time steps with lower $\overset{-}{r}$ occurred between 12.00–14.00 and 18.00–20.00 across the four cruise sailings (electronic supplementary material, figure S1). Passengers were likely to be engaged in dining during these periods, and previous work showed that dining settings promote social contact [11]. The seating arrangements or the movement patterns (e.g. buffet counters) facilitate increased mixing and interaction between passengers of different cabins (figure 3*b* and electronic supplementary material, figure S1). High values of $\overset{-}{r}$ were also observed at the start and end of each day, the result of contact between passengers from the same cabin.

Pre-pandemic community networks from the United Kingdom exhibited an even higher $\overset{-}{r}$ of 0.73 (IQR 0.65−0.81). About 40% of thecontacts occurred before 09.00 and after 17.00 when the individual is likely to be at home with household contacts (figure 3*a*,*b*, and electronic supplementary material, figure S1). In contrast, networks from schools, hospitals and workplaces showed lower $\overset{-}{r}$ of 0.58 (IQR 0.44−0.69), 0.49 (IQR 0.36−0.64) and 0.50 (IQR 0.33−0.61), respectively. In these networks, $\overset{-}{r}$ was below 0.5 for about half of the observed duration and changes in $\overset{-}{r}$ did not exhibit any time trends, unlike the cruise or community networks (electronic supplementary material, figure S1). Moreover, at low and high values of $\overset{-}{r}$, there was no apparent variation in the type of retained contacts. We estimated that contacts made between classmates or individuals of the same department form the majority of the contacts in each time step for the high school network, about 60% for the hospital network and 80% for the workplace networks. We observed similar proportions among the retained contacts (figure 3*b*).

The overall patterns in our analysis remained unchanged when we performed sensitivity analyses around choice of time step and contact definition. We obtained similar results when assuming undirected contacts in the non-cruise settings (electronic supplementary material, figure S2), although when using fixed time steps of 15 min or 1 h for all networks, the overall median r of all networks was slightly lower than the main analysis. However, $\overset{-}{r}$ in both the cruise and community networks remained higher than networks from schools, hospitals and workplaces (electronic supplementary material, figures S3 and S4).

Across all networks, the distribution of contacts (i.e. degree distribution) over the study period was different due to different population sizes and duration of observation and did not exhibit a power law distribution (electronic supplementary material, figure S5). On the contrary, the distribution of contacts in each time step was similar across all networks with most individuals having less than five contacts (electronic supplementary material, figure S6). In general, as the number of contact episodes made by an individual increased, the cumulative duration of contact aggregated over all episodes increased (electronic supplementary material, figure S7).

### 3.2. Epidemiological metrics

**Figure 4.**
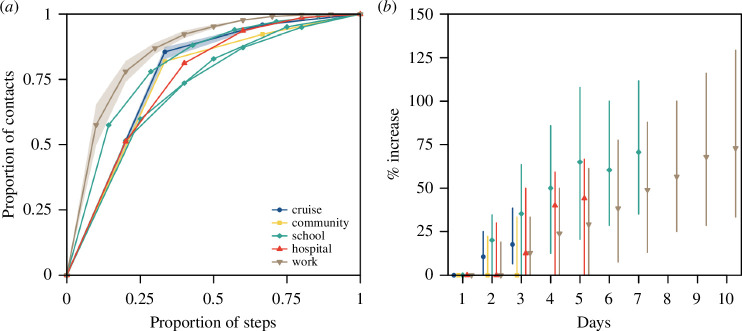
Contact pairs over the study duration in different networks, (*a*) cumulative distribution of contact encounters in days in pairs of contact. Study duration varied across networks and was normalized. For networks with the same study duration, such as the four cruises and three workplace networks, the distribution was represented by the median (lines) and range (shaded region). For networks with different study durations, such as the three high school networks, or a single network study, such as the community and hospital networks, the distribution of each network study was illustrated, (*b*) median (shapes) and range (lines) of the relative difference in the number of unique contacts.

Although a longer study duration will, in theory, increase the probability of observing repeated contact over multiple days, there was some agreement across different networks on the proportion of total measured contacts that occurred in 1 day out of all days in respective network studies. For studies conducted over 3 days, the proportion of total contacts that occurred over 1 day was 86% (range 83%−87%) in the cruises and 82% in the community (figure 4*a*). For studies conducted over longer durations of up to 10 days of recorded contacts, the proportion of total contacts recorded in a given day was 57% (range 52%−60%) in the high schools, 51% in the hospital and 47% (range 38%−55%) in the workplace networks (figure 4*a*). Across all the networks, over 75% of the contacts either occurred over 1 day only or were repeated for less than half the study duration (figure 4*a*).

When planning outbreak control measures such as contact tracing, we need to consider the number of unique contacts made per infected individual. If we did not account for repeated contacts over the days and instead assumed the measured number of daily contacts would be made independently each day, we could overestimate the number of unique contacts. With the exception of the community network, we found that we would overestimate the unique contacts by 13%−35% across all networks after 3 days of observation under this independence assumption (figure 4*b*). For longer study duration in the schools, this difference between the total and unique contacts was 71% (IQR 35%−110%) after 7 days; for workplaces, the difference rose to 73% (IQR 33%−130%) after 10 days (figure 4*b*).

### 3.3. Extent of superspreaders and superspreading events

**Figure 5.**
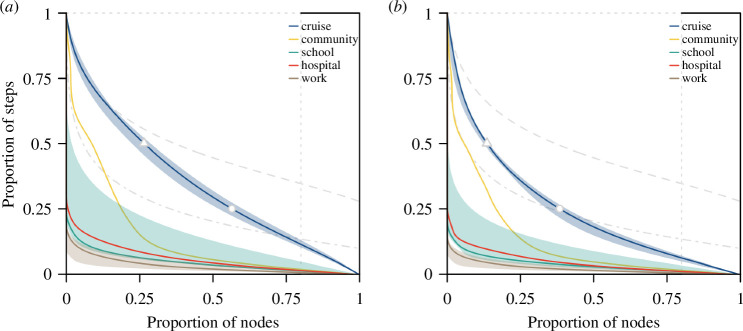
Proportion individuals who are potential ‘superspreaders’ or drive superspreading events in respective networks, estimated based on (*a*) contact episodes or (*b*) contact duration. For reference, grey lines represent homogeneous static network (dotted), homogeneous dynamic network in 25 time steps (dashed) and overdispersed dynamic network in 10 time steps (dot dashed). Cut-off marks for the proportion of individuals in the cruise networks who were highly connected for more than half the total number of time steps (triangle) and those who were highly connected for less than a quarter of the time (dot) as shown.

Depending on the level of overdispersion of individual-level contacts in a network and the duration of observation, our ability to correctly predict highly connected individuals in a given time period will vary. For a homogeneous static network, 80% of the population accounts for 80% of the contacts made. As such, 80% of the population would be identified as highly connected across all the time steps, while the remaining 20% of the population would never be identified in this group (figure 5, dotted lines). For a fully dynamic homogeneous network with 25 time steps, 80% of the population accounts for 80% of the contacts in each time step. Given changes in the network structure over the time steps, only 40% of the population would be identified for at least half the total number of time steps. For a fully dynamic overdispersed network with 10 time steps, 50% of the population accounts for 80% of the contacts in each time step. Consequently, only 5% of the population would be identified in at least half the observations. We found that as networks transition from homogeneous to overdispersed, and as the duration of observation increases, the proportion of highly connected individuals that can be identified consistently is reduced.

Real-world networks with higher levels of contact retention had a higher probability of correctly predicting frequent, highly connected individuals, but these individuals only accounted for less than 30% of the population. These are individuals who account for the top 80% of the contact episodes for at least half of the number of observed time steps (i.e. potential superspreaders, top left region of each panel in figure 5). A convex curve that is further away from the *y*-axis in figure 5 implies that individuals who are highly connected in one time step are very likely to be highly connected in other time steps, thereby increasing our ability to predict superspreaders as compared with another convex curve that is closer to the *y*-axis.

In real-world cruise contact networks, 26% (range 22%−29%) of the population were predicted to fall into this ‘potential superspreader’ category (i.e. top left region where the proportion of nodes is 0–0.26, and the proportion of time steps is 0.5–1). The remaining population are individuals who have high connections but for short periods of time only. These are individuals who are likely to drive superspreading events (i.e. bottom right region of each panel in figure 5). In particular, 44% (range 40%−48%) of the population were identified for less than a quarter of the observed time steps (figure 5*a*, bottom right region where the proportion of nodes is 0.56–1 and the proportion of time steps is 0–0.25). In the community network, 9% of the population would be predicted to be potential superspreaders, while 81% of the population are likely to drive superspreading events for less than a quarter of the time and 10% fall into neither category (i.e. the region between superspreader and superspreading events; figure 5*a*). The proportion of the population identified as potential superspreaders was less than 5% in the high school, hospital and workplace networks; the majority of the individuals would, if anything, drive superspreading events instead (figure 5*a*). Similar trends were observed when analysing the proportion of the population that accounted for the top 80% of the contact duration (figure 5*b*).

In the above main analysis to identify the extent of superspreaders and superspreading events, the time steps for each network were based on the median delay between contacts (table 1). The duration of the time step was short and ranged between 100 s and 15 min. In a sensitivity analysis, we identified the nodes that accounted for the top 80% of the contact episodes for a time step lasting for 1 day and estimated the proportion of nodes that were consistently identified to be highly connected over the study period (electronic supplementary material, figure S8). The trends of our findings remained unchanged across different time step assumptions.

## 4. Discussion

Using real-world contact data collected from a variety of settings over different days and population sizes, we assessed the key structural properties of temporal networks that drive transmission processes and, hence, influence the effectiveness of outbreak control measures. We estimated that most individuals in each social context had high levels of connectivity with others for less than a quarter of the study duration. Contact retention and the type of contacts driving this retention varied across settings, emphasizing the need for tailored outbreak analysis and control strategies for different settings.

In our analysis, we compared the properties of the real-world temporal networks relative to static and fully dynamic networks, normalized by the population size. This enabled us to contextualize our findings and allow for appropriate comparison across different networks. In particular, our study highlighted an inherent difficulty in predicting superspreaders over time across different settings [6]. In cruise data, the high level of consistency in identifying highly connected individuals (i.e. 26% of the population identified to account for the top 80% of the contacts in more than half the total observed time steps) was probably influenced by the prevailing COVID-19 restrictions on-board during the study. Passengers and crews were encouraged to remain within their travel or working groups and to practise physical distancing from other groups [11]. However, the level of consistency in identifying highly connected individuals was generally low in all other networks. More than 80% of the population was identified to be highly connected for only a short period of the study duration. Targeting small groups of infectious individuals with high levels of connectivity has been shown to, in theory, produce an effective and efficient reduction in transmission, but such studies were largely based on static networks [23,24]. In contrast, our study showed that if we were to sample a network for a few days or a short period of time and target individuals with high measured connectivity, the level of connectivity in the same individuals would generally turn out to be much lower if data collection was to be repeated in the near future. As such, when designing interventions to identify potential ‘superspreaders’, we would need to target a greater number of individuals than basic theory from static networks suggests in order to achieve the same reduction in transmission.

When an outbreak occurs, outbreak control policies often target subpopulations rather than individuals given the lack of information on contact patterns [15]. Across most social settings we analysed, contacts between individuals in the same social group (e.g. same cabin, department or school class) dominated interactions, even if retention of these contacts was variable. For high schools and workplaces, we estimated low contact retention even when most of these contacts were formed between individuals of the same class. This result corroborates previous findings indicating low levels of repeated contact among household contacts for those residing in dormitories [14].

When implementing outbreak control policies, our results suggest it is important to consider if the priority is to reduce disease introductions, or reduce transmission if introduced to a locality, and thus, which are the appropriate individuals or subpopulations to target with restrictions. In schools and workplaces, the majority of close contacts were from individuals of the same department or class, implying that targeted rather than school- or workplace-wide closures could still help to minimize disruption to activities. This would be particularly relevant if disease prevalence in the wider population is low and the likelihood of introductions to other departments or classes is low. In contrast, for settings such as hospitals, contacts from both the same (e.g. nurse–nurse contacts) or different (e.g. patient–nurse contacts) departments are likely to be retained over consecutive time steps. This higher proportion of contacts between different departments is expected given the multifaceted roles of healthcare workers [18]. Thus, more stringent measures to reduce the risk of nosocomial outbreaks starting are highly important to avoid disruptions to hospital functions.

While the use of detailed contact data to plan quarantine measures can provide an upper limit on the resources required [7,9], our results suggest the occurrence of repeated contacts would mean that simple analysis, based on cross-sectional data collection that assumes independence of contacts, would generally overestimate the resources required for contact tracing each case. With the occurrence of pre-symptomatic transmission for SARS-CoV-2 [25,26] and delays from symptoms onset to testing to isolation [27,28], contact tracing would involve the identification of cases over 3−11 days, and repeated contacts arising from regular daily activities would imply that the actual contacts made over this period are 20%−70% lower than the sum of all the contact episodes recorded independently on each day. Simple compartmental models assume homogeneous mixing (i.e. a constant number of contacts but contacts are randomized each day), while branching process models simulate onward transmission based on a number of contacts. Both types of models typically do not account for repeated contacts and will lead to an overestimation of the outbreak size. In contrast, static network models do not allow for infectors to form new contacts and seed outbreaks in other parts of the network. This causes the outbreak to end prematurely. While more studies are required to further characterize temporal networks, these simplified models would help to establish the lower and upper limits for resource planning.

There are some limitations to our study. First, we focused on the network and epidemiological metrics between pairs of contacts (e.g. duration and type of contacts). We did not study the changes in clustering on temporal networks and overlay the dynamics of host-related infectiousness profiles and factors (e.g. age and gender) on these networks. As such, this limits our ability to make conclusions on the impact of temporal contacts on outbreak size, time to outbreak extinction and herd immunity thresholds. Nevertheless, the current study is a first step in characterizing temporal networks. Our ‘retention index’, $\overset{-}{r}$, quantifies the retention of contacts in temporal networks relative to static and highly dynamic networks. We analysed the type of contact pairs that are likely to be retained and highlighted the implications to control measures. Future studies could extend this metric to account for higher order network properties. This would allow us to better understand the impact of time-varying contacts on disease transmission and study the feasibility of using simpler static networks or compartmental models. Furthermore, while there was considerable individual-level variation in viral shedding profiles during the COVID-19 pandemic [29,30], one study showed that the heterogeneity in generating secondary cases was better explained by the heterogeneity in contact patterns [30]. This was because the probability of infection saturates beyond an exposed viral load [31] and instead, the number of contacts was the main dominant driver of the overall number of plausible infections—a key focus of our study.

Second, different devices were used to measure the networks in different studies. They could either detect face-to-face interactions or RFID signals from all directions. As each device has a different calibration, the measured differences between the networks can be an outcome of the data collection process or due to inherent differences in the context setting. As such, in the main analysis, we defined the contact duration and delay between contacts based on the characteristics of each network (table 1). In our sensitivity analysis, we standardized the duration and delay. The changes in $\overset{-}{r}$ for different networks were similar in both analyses. Hence, the impact of the device setting on the overall observed contact patterns was not expected to be significant.

Third, real-life contact typically exists in an open population, and thus, not every contact was captured in these network studies. If these missed contacts were to occur in specific subpopulations this may result in a shift in the proportion of retained contact types. Furthermore, the level of connectivity in missed contacts is unknown. As such, our analysis could over- or under-estimate the proportion of superspreaders and superspreading events. However, our findings would remain valid if we assume that the missingness is independent of the level of connectivity and can occur in any subpopulation.

Our analysis highlights the difficulty in identifying highly connected individuals unless real-world contacts are surveyed at high resolution over several days. However, we did find more consistency in contact patterns among specific settings and social groups. Hence, outbreak control measures that target key settings or at-risk subpopulations are likely to be more effective than targeting specific individuals if currently available data approaches continue to be used. Comparing the dynamics of such real-world temporal networks and corresponding outbreak data would further advance our understanding of the risk of different contacts in practice.

## Data Availability

Data and code available at [32]. Supplementary material is available online [33].

## References

[1] Keeling MJ, Eames KTD. 2005 Networks and epidemic models. J. R. Soc. Interface **2**, 295–307. (10.1098/rsif.2005.0051) 16849187 PMC1578276

[2] Newman MEJ. 2002 Spread of epidemic disease on networks. Phys. Rev. E **66**, 016128. (10.1103/PhysRevE.66.016128) 12241447

[3] Grassly NC, Fraser C. 2008 Mathematical models of infectious disease transmission. Nat. Rev. Microbiol. **6**, 477–487. (10.1038/nrmicro1845) 18533288 PMC7097581

[4] Keeling MJ, Rohani P. 2008 Modelling infectious diseases in humans and animals. Princeton, NJ: Princeton University Press.

[5] Holme P, Saramäki J. 2012 Temporal networks. Phys. Rep. **519**, 97–125. (10.1016/j.physrep.2012.03.001)

[6] Eames K, Bansal S, Frost S, Riley S. 2015 Six challenges in measuring contact networks for use in modelling. Epidemics **10**, 72–77. (10.1016/j.epidem.2014.08.006) 25843388

[7] Kucharski AJ *et al*. 2020 Effectiveness of isolation, testing, contact tracing, and physical distancing on reducing transmission of SARS-CoV-2 in different settings: a mathematical modelling study. Lancet Infect. Dis. **20**, 1151–1160. (10.1016/S1473-3099(20)30457-6) 32559451 PMC7511527

[8] Tupper P, Boury H, Yerlanov M, Colijn C. 2020 Event-specific interventions to minimize COVID-19 transmission. Proc. Natl Acad. Sci. USA **117**, 32038–32045. (10.1073/pnas.2019324117) 33214148 PMC7749284

[9] Koo JR, Cook AR, Park M, Sun Y, Sun H, Lim JT, Tam C, Dickens BL. 2020 Interventions to mitigate early spread of SARS-CoV-2 in Singapore: a modelling study. Lancet Infect. Dis. **20**, 678–688. (10.1016/S1473-3099(20)30162-6) 32213332 PMC7158571

[10] Cattuto C. 2023 SocioPatterns. See http://www.sociopatterns.org/datasets (accessed 9 August 2023).

[11] Pung R *et al*. 1956 Using high-resolution contact networks to evaluate SARS-CoV-2 transmission and control in large-scale multi-day events. Nat. Commun. **13**. (10.1038/s41467-022-29522-y) PMC9005731 35414056

[12] Kissler SM, Klepac P, Tang M, Conlan AJK, Gog JR. 2020 Sparking 'the BBC four pandemic': leveraging citizen science and mobile phones to model the spread of disease. bioRxiv 479154. (10.1101/479154)

[13] Wymant C *et al*. 2021 The epidemiological impact of the NHS COVID-19 app. Nature **594**, 408–412. (10.1038/s41586-021-03606-z) 33979832

[14] Read JM, Eames KTD, Edmunds WJ. 2008 Dynamic social networks and the implications for the spread of infectious disease. J. R. Soc. Interface **5**, 1001–1007. (10.1098/rsif.2008.0013) 18319209 PMC2607433

[15] Eames KTD, Read JM, Edmunds WJ. 2009 Epidemic prediction and control in weighted networks. Epidemics **1**, 70–76. (10.1016/j.epidem.2008.12.001) 21352752

[16] Mastrandrea R, Fournet J, Barrat A. 2015 Contact patterns in a high school: a comparison between data collected using wearable sensors, contact diaries and friendship surveys. PLoS One **10**, e0136497. (10.1371/journal.pone.0136497) 26325289 PMC4556655

[17] Fournet J, Barrat A. 2014 Contact patterns among high school students. PLoS One **9**, e107878. (10.1371/journal.pone.0107878) 25226026 PMC4167238

[18] Vanhems P, Barrat A, Cattuto C, Pinton JF, Khanafer N, Régis C, Kim B a, Comte B, Voirin N. 2013 Estimating potential infection transmission routes in hospital wards using wearable proximity sensors. PLoS One **8**, e73970. (10.1371/journal.pone.0073970) 24040129 PMC3770639

[19] Génois M, Vestergaard CL, Fournet J, Panisson A, Bonmarin I, Barrat A. 2015 Data on face-to-face contacts in an office building suggest a low-cost vaccination strategy based on community linkers. Netw. Sci. **3**, 326–347. (10.1017/nws.2015.10)

[20] Génois M, Barrat A. 2018 Can co-location be used as a proxy for face-to-face contacts? EPJ Data Sci. **7**, 1–18. (10.1140/epjds/s13688-018-0140-1)

[21] Yusef D *et al*. 2020 Large outbreak of coronavirus disease among wedding attendees, Jordan. Emerging Infect. Dis. **26**, 2165–2167. (10.3201/eid2609.201469) PMC7454095 32433907

[22] Siddique H. 2020 'Super-spreader' brought coronavirus from Singapore to Sussex via France. The Guardian. See https://www.theguardian.com/world/2020/feb/10/super-spreader-brought-coronavirus-from-singapore-to-sussex-via-france.

[23] Woolhouse MEJ *et al*. 1997 Heterogeneities in the transmission of infectious agents: implications for the design of control programs. Proc. Natl Acad. Sci. USA **94**, 338–342. (10.1073/pnas.94.1.338) 8990210 PMC19338

[24] Lloyd-Smith JO, Schreiber SJ, Kopp PE, Getz WM. 2005 Superspreading and the effect of individual variation on disease emergence. Nature **438**, 355–359. (10.1038/nature04153) 16292310 PMC7094981

[25] Wei WE, Li Z, Chiew CJ, Yong SE, Toh MP, Lee VJ. 2020 Presymptomatic transmission of SARS-CoV-2 – Singapore, January 23–March 16, 2020. MMWR Morb. Mortal. Wkly. Rep. **69**, 411–415. (10.15585/mmwr.mm6914e1) 32271722 PMC7147908

[26] Ferretti L, Wymant C, Kendall M, Zhao L, Nurtay A, Abeler-Dörner L, Parker M, Bonsall D, Fraser C. 2020 Quantifying SARS-CoV-2 transmission suggests epidemic control with digital contact tracing. Science **368**, eabb6936. (10.1126/science.abb6936) 32234805 PMC7164555

[27] Ali ST, Wang L, Lau EHY, Xu XK, Du Z, Wu Y, Leung GM, Cowling BJ. 2020 Serial interval of SARS-CoV-2 was shortened over time by nonpharmaceutical interventions. Science **369**, 1106–1109. (10.1126/science.abc9004) 32694200 PMC7402628

[28] Bi Q *et al*. 2020 Epidemiology and transmission of COVID-19 in 391 cases and 1286 of their close contacts in Shenzhen, China: a retrospective cohort study. Lancet Infect. Dis. **20**, 911–919. (10.1016/S1473-3099(20)30287-5) 32353347 PMC7185944

[29] Russell TW *et al*. 2024 Combined analyses of within-host SARS-CoV-2 viral kinetics and information on past exposures to the virus in a human cohort identifies intrinsic differences of Omicron and Delta variants. PLoS Biol. **22**, e3002463. (10.1371/journal.pbio.3002463) 38289907 PMC10826969

[30] Quilty BJ *et al*. 2024 Disentangling the drivers of heterogeneity in SARS-CoV-2 transmission from data on viral load and daily contact rates. medRxiv. (10.1101/2024.08.15.24311977) PMC13552949 42664259

[31] Zhang X, Wang J. 2021 Dose-response relation deduced for coronaviruses from coronavirus disease 2019, severe acute respiratory syndrome, and Middle East respiratory syndrome: meta-analysis results and its application for infection risk assessment of aerosol transmission. Clin. Infect. Dis. **73**, e241–e245. (10.1093/cid/ciaa1675) 33119733 PMC7665418

[32] Pung R, Kucharski A, Firth J, Russell TW, Rogers T, Lee V. 2024 rachaelpung/temporal_networks: [Data set]. In journal of the royal society interface. Zenodo. (10.5281/zenodo.14032234)39689845

[33] Pung R, Firth JA, Russell T, Rogers T, Lee VJ, Kucharski A. 2024 Supplementary material from: Temporal contact patterns and the implications for predicting superspreaders and planning of targeted outbreak control. Figshare. (10.6084/m9.figshare.c.7569546)39689845

